# Potential value of cartilage and bone soluble biomarkers in evaluating joint damage in juvenile idiopathic arthritis

**DOI:** 10.1186/1546-0096-12-S1-P19

**Published:** 2014-09-17

**Authors:** Silvia Pederzoli, Giuliana Cangemi, Angela Pistorio, Cinzia Gatti, Giulia Consiglieri, Marta Dellepiane, Diliana Beleva, Antonella Buoncompagni, Alberto Martini, Clara Malattia

**Affiliations:** 1 Pediatria II, Italy; 2 U.O: Laboratorio Centrale di Analisi. Laboratory Unit, Italy; 3 Servizio di epidemiologia e biostatistica, I. G. Gaslini, Italy; 4 Dipartimento di Scienze Pediatriche, Università degli Studi di Genova, Genova, Italy

## Introduction

Serum biomarkers of bone and cartilage turnover were found to predict structural damage progression in Rheumatoid Arthritis (RA). Their potential value in Juvenile Idiopathic Arthritis (JIA) has never been explored.

## Objectives

1) To examine associations between soluble biomarkers of bone (CTX-I) and cartilage degradation (C1-2C, C2C) and joint damage as assessed by Conventional Radiography (CR) and Magnetic Resonance Imaging (MRI) in patients with JIA. 2) To investigate whether these biomarkers can predict structural damage progression.

## Methods

The clinically more affected wrist of 88 JIA patients was studied with CR according to adapted Sharp/van der Heijde score (SHS) method and MRI by using the OMERACT RA-MRI-score, coupled with standard clinical assessment. One-year CR follow-up was available in 65 patients, whereas one-year MRI follow-up was available in 51 patients. Serum CTX-I, C1-2C, C2C were measured by ELISA assays in all patients at the enrollment and in gender- and age-matched healthy controls (N= 154).

## Results

Unlike adults with RA, CTX-I, C1,2C and C2C levels were significantly lower in JIA patients than healthy controls (P< 0.0001).

Biomarker levels did not correlate with clinical measure of disease activity and damage, disease duration and with Sharp/van der Heijde score (SHS), and RAMRIS bone erosion score.Median C1,2C and C2C were significantly higher in patients with structural damage progression according to JSN (joint space narrowing)-SHS score compared to patients without progression (C1,2C: 240 ng/ml vs 125 ng/ml, P=0.01; C2C: 133.7 ng/ml vs 65.7 ng/ml, P= 0.001). Unlike RA, patients with radiographic progression showed significantly lower levels of CTX-I (1.03 ng/ml) compared patients without structural damage progression (1.53 mg/ml P=0.03).Patients, who required either initiation of methotrexate or addition of a biologic agent at the 6 months follow-up visit, had significantly higher levels of C1,2C (P=0.027) and C2C (P=0.034) compared to patients who did not require treatment changes.

## Conclusion

Our results suggest an inhibition of bone and cartilage turnover in patients with JIA. Biomarkers of cartilage degradation are promising as potential predictors of structural damage progression and severity of disease course.

## Disclosure of interest

None declared.

